# Proteoglycans as Regulators of Receptor Trafficking and Spatial Signaling in Cancer

**DOI:** 10.3390/cancers18172832

**Published:** 2026-09-01

**Authors:** Aikaterini Berdiaki, Maria Konstantaraki, Zuha Ajlan, Maria Marmara, Aristidis Tsatsakis, George Tzanakakis, Dragana Nikitovic

**Affiliations:** 1 Department of Histology-Embryology, Medical School, University of Crete, 70013 Heraklion, Greece; berdiaki@uoc.gr (A.B.); medp2012170@med.uoc.gr (M.K.); imed114@edu.med.uoc.gr (Z.A.); marmaramaria25@gmail.com (M.M.); tzanakak@med.uoc.gr (G.T.); 2 Dermatology Department, University Hospital of Heraklion, 71500 Iraklio, Greece; 3 Center of Toxicology & Science Applications, Medical School, University of Crete, 71003 Heraklion, Greece; tsatsaka@uoc.gr; 4 Dermatology Department, University Hospital of Heraklion, Voutes Campus, University of Crete, 70013 Heraklion, Greece; 5 Biomedical Science and Technology Park, I.M. Sechenov First State Medical University, 119991 Moscow, Russia

**Keywords:** proteoglycans, receptor trafficking, receptor endocytosis, spatial signaling, extracellular matrix, glycocalyx, receptor tyrosine kinases, glycosaminoglycan, cancer signaling, tumor microenvironment

## Abstract

Cellular responses depend on the coordinated regulation of receptor activation, trafficking, and signaling across distinct membrane and intracellular compartments. Proteoglycans (PGs), traditionally regarded as structural components of the extracellular matrix and co-receptors, are now emerging as active organizers of receptor dynamics. This review examines the mechanisms through which PGs regulate receptor trafficking and spatial signaling in cancer. Recent studies addressing the roles of proteoglycans in receptor clustering, endocytic pathway selection, intracellular trafficking, recycling, lysosomal degradation, endosomal signaling, exosome biogenesis, and nuclear receptor transport were evaluated, with particular attention to the strength of direct evidence for trafficking. Particular attention was given to the complementary functions of proteoglycan core proteins, glycosaminoglycan chains, and extracellular matrix remodeling in shaping receptor behavior. Current evidence demonstrates that proteoglycans govern multiple stages of receptor biology by controlling ligand presentation, receptor accessibility, membrane organization, intracellular trafficking, and signaling persistence. Individual proteoglycans exert distinct effects on receptor fate, ranging from receptor downregulation and degradation to sustained endosomal signaling and nuclear receptor translocation. Cancer-associated alterations in proteoglycan expression, glycosaminoglycan sulfation, heparanase activity, ectodomain shedding, and glycocalyx organization remodel signaling networks, strengthen communication between tumor and stromal cells, and promote tumor progression, immune modulation, metastasis, and therapeutic resistance. Proteoglycans have emerged as fundamental regulators of receptor trafficking and signaling architecture. Through the integration of extracellular matrix organization with receptor dynamics, they shape the spatial and temporal properties of oncogenic signaling. This conceptual framework broadens our understanding of tumor biology and identifies proteoglycan-dependent regulation of receptor trafficking as a promising avenue for future therapeutic intervention.

## Simple Summary

Proteoglycans are complex molecules located on the cell surface and within the extracellular matrix that have traditionally been viewed as structural components or co-receptors. However, growing evidence shows that they actively regulate where receptors are located, how they move inside cells, and how long signaling persists. These processes are particularly important in cancer, where abnormal receptor trafficking contributes to uncontrolled growth, invasion, metastasis, and resistance to therapy. In this review, we summarize current knowledge on how proteoglycans regulate receptor clustering, endocytosis, intracellular trafficking, recycling, degradation, endosomal signaling, exosome biology, and nuclear receptor transport. We also discuss how alterations in proteoglycan expression, glycosaminoglycan composition, and extracellular matrix remodeling reshape signaling networks within the tumor microenvironment. Understanding these mechanisms identifies proteoglycans as central organizers of cancer signaling and highlights new opportunities for therapeutic intervention targeting receptor trafficking and spatial signaling regulation.

## 1. Introduction

Proteoglycans (PGs) are components of the extracellular matrix (ECM), expressed in most tissue types, together with glycosaminoglycans (GAGs), collagens, laminins, fibronectin, elastin, other glycoproteins and proteinases [1]. ECM, most importantly, does not only exist as a supporting mechanical environment, but it is also a source of regulating factors that affect specific signaling pathways inside the cells [2,3]. PGs have been described as part of cells’ mechanosensing and mechanotransduction pathways [4]. ECM interaction with cells begins with the interaction of its components with the cell membrane or intracellular receptors. Receptor activation initiates intracellular signaling cascades regulating gene expression [5].

### 1.1. From Ligand Availability to Spatial Control of Signaling

Receptor action is modulated by ligand availability and spatial (lateral and axial) localization. Lateral spatial regulation refers to the organization and clustering of receptors within distinct plasma membrane microdomains, whereas axial spatial regulation involves receptor trafficking between the plasma membrane and intracellular compartments, including endosomes, lysosomes and the nucleus [6,7]. Receptor trafficking determines the location of action at the different compartments of the cell, the duration of signaling and the specificity of signaling outputs [8,9]. Moreover, receptor signaling is not limited to the cell surface, but can continue within the endocytic compartment, comprising the axial spatial control of cell signaling transduction [7].

Furthermore, it is important to note that receptors do not function as individual units; they form clusters that interact with multiple molecules, adding complexity to their activation and signaling [6,10,11,12,13]. Advantages of receptor clustering include restricted diffusion of receptors at the membrane and amplification of signal by multiple receptors’ activation [6]. In this context, membrane nanodomains and lipid rafts are increasingly recognized as specialized signaling platforms that regulate receptor accessibility, internalization, and signaling persistence [14].

Diverse receptors have been described in mammalian cells. An important family is the receptor tyrosine kinases (RTKs), which have been shown to modulate cellular functions such as proliferation, migration, adhesion, differentiation, and survival [15]. Additionally, receptors interact with endocytic adaptor proteins, which enhance their internalization into the endocytic trafficking network, thus inducing their removal from the cell surface. The latter, receptor internalization, is now considered an essential part of signaling, controlling the strength and spatial and temporal restrictions to RTK signals [16,17]. Thus, endocytosis is no longer viewed solely as a mechanism for receptor degradation, but also as an active regulator of signaling specificity and duration [9]. Altered receptor trafficking, aberrant recycling, and sustained endosomal signaling have been increasingly associated with tumor progression and therapeutic resistance [18,19].

G protein-coupled receptors (GPCRs) are another example of transmembrane receptors for which membrane trafficking has been shown to modulate their action. GPCR trafficking seems to involve organized plasma membrane signaling microdomains, formed by distinct endocytic and exocytic pathways, and the spatial control of endomembrane localization [20]. Studies have demonstrated that trafficking of GPCRs affects heterotrimeric G protein signaling patterns [21]. Divergent sorting of GPCRs after ligand-induced endocytosis to either recycling or degradative/lysosomal pathways essentially produces opposite effects on receptor signaling via their cognate G proteins [8,21].

An example of adhesion receptors that associate with both ECM molecules and surface ligands as well as the actin cytoskeleton in the cytoplasm, through linker proteins, is integrins [22,23]. Integrin polypeptide family members combine to form heterodimeric receptors aiding in the complexity of action [22,23]. Integrins enable cells to sense the topology and mechanochemical properties of their ECM and respond by altering their position and differentiation stage [22,23]. Consequently, receptor signaling is tightly connected not only to ligand availability but also to the biomechanical and topological properties of the surrounding ECM microenvironment.

### 1.2. Deregulation of Receptor Action in Cancer and the Possible Role of Proteoglycans

Deregulation of receptor expression, activation, and trafficking is a common feature of cancer and can contribute to sustained proliferative signaling, invasion, metastatic progression, and therapeutic resistance. Prominent examples include aberrant signaling through EGFR and other ErbB family members, MET, IGF-IR, and VEGFRs, as well as altered integrin-dependent signaling. Receptor-mediated signaling therefore contributes to multiple stages of tumor development and has become an important target for anticancer therapy [7,19,24].

Possible receptor signaling modulators are PGs, which are known to interact with different types of receptors, affect cellular organization, adhesion, signal transduction, and most importantly, cancer progression. PGs comprise a protein core and one or more covalently linked GAG chains [1,25,26,27]. PGs are categorized into cell surface PGs, which can either be transmembrane or glycosylphosphatidylinositol-anchored PGs [28,29]. The next category includes pericellular and extracellular PGs, which are secreted into the ECM and form complexes with a plethora of molecules [26,30,31]. Finally, there is just one intracellular PG member [26,32,33].

PGs within the ECM contribute to the formation of growth factor pools and protect bound growth factors from proteolytic degradation, thereby establishing gradients that regulate cellular signaling [29,34]. Additionally, PGs stabilize ligand-to-receptor binding, activate signaling cascades, or help attenuate signaling of other growth factor receptors [29,34,35]. Through both their core proteins and GAG chains, PGs also regulate receptor clustering, ligand presentation, receptor internalization, and signaling compartmentalization [25,29,34,36]. Indeed, transmembrane PGs, syndecans (SDCs) have been found to participate in the recycling of adhesion and growth factor receptors by trapping receptors through their glycosaminoglycan chains [37].

PGs are increasingly recognized as active regulators of receptor signaling organization, rather than merely structural components of the ECM or passive co-receptors. By modulating ligand sequestration, receptor accessibility, membrane compartmentalization, trafficking and endosomal signaling, PGs influence where, how long and through which intracellular routes signaling persists. This emerging concept mechanistically links ECM remodeling, glycocalyx organization, receptor trafficking, mechanotransduction, and tumor-associated reconfiguration of signaling networks during cancer progression and therapeutic resistance [4]. In this review, PGs are discussed as active organizers of signaling networks that shape the spatial organization and persistence of receptor signaling.

This narrative review was based on literature identified through searches of PubMed, Scopus, and Web of Science, using combinations of the terms “proteoglycan,” “glycosaminoglycan,” “receptor trafficking,” “receptor internalization,” “endocytosis,” “recycling,” “lysosomal degradation,” “endosomal signaling,” “nuclear trafficking,” “cancer,” and individual proteoglycan names. Priority was given to original mechanistic studies directly examining receptor localization, internalization, recycling, degradation, or intracellular redistribution, complemented by relevant studies addressing receptor-associated signaling, extracellular matrix remodeling, glycocalyx organization, and therapeutic targeting. Studies from non-malignant systems were included when they provided mechanistic information not yet available in cancer models and are identified as such in the text. The literature search was updated through August 2026.

## 2. Proteoglycans as Spatial Organizers of Receptor Signaling

As mentioned above, PGs exhibit a modular architecture that enables them to function as dynamic regulators of receptor organization, trafficking, and spatial signaling. PGs consist of a protein core covalently linked to one or more GAG chains. GAGs are linear polysaccharides present in five different types: chondroitin sulfate (CS), dermatan sulfate (DS), keratan sulfate (KS), heparan sulfate (HS) and heparin (Hep) [1,25,26,27,28,29,38,39]. The diversity in GAG structure, as well as differences in PG amino acid sequence, size, shape, and the number of attached GAG chains, gives PGs a structural complexity that can modulate normal tissue physiology and cancer progression [1,25,26,27,38,39]. Variability in GAG composition, sulfation patterns and core protein structure contributes substantially to ligand specificity, receptor interactions and signaling regulation [1,26,38]. Indeed, their structure integrates exquisite molecular specificity with biochemical adaptability. Through this dual organization, PGs exhibit high competence in coordinating receptor–ligand interactions, receptor clustering, and intracellular trafficking, thereby fine-tuning and shaping the initiation, duration, and compartmentalization of signals across cellular domains.

In cancer, changes in PG expression are accompanied by alterations in GAG composition and sulfation, together with remodeling of the glycocalyx and ECM. These changes modify ligand availability, receptor accessibility, and the spatial organization of signaling within the tumor microenvironment [4,40,41].

### 2.1. Core Protein- and GAG-Mediated Regulation of Receptor Signaling

A central feature of PG function is the interplay between core protein domains and GAG chains, highlighting their combined importance in regulating receptor dynamics at multiple levels. Indeed, core proteins and GAG chains frequently cooperate to regulate receptor accessibility, ligand presentation, receptor clustering and downstream signaling specificity.

The core proteins of SLRPs possess intrinsic receptor-binding capacity and can directly modulate receptor activity and fate. This has been most extensively demonstrated for decorin, which directly interacts with several RTKs, including EGFR and Met [42,43,44]. Decorin binding to EGFR is mediated by its protein core [43], while subsequent studies demonstrated receptor internalization and degradation [45]. Indeed, the binding of decorin to EGFR initiates transient receptor phosphorylation followed by caveolin-mediated endocytosis and lysosomal degradation, resulting in sustained suppression of MAPK/ERK signaling and inhibition of tumor cell proliferation [45]. Similarly, decorin engagement of Met leads to receptor downregulation and inhibition of β-catenin signaling, suppressing tumor cell migration and invasion [44]. In endothelial cells, decorin interaction with vascular endothelial growth factor receptor-2 (VEGFR2) activates PEG3-dependent autophagy and angiostatic signaling, linking receptor regulation to inhibition of tumor angiogenesis [46], whereas in breast carcinoma decorin initiates cell mitophagy via mitostatin by interacting with VEGFR2 or the MET receptor tyrosine kinase [47]. Together, these findings indicate that decorin, via its core protein, can regulate receptor activation, trafficking, and signaling duration.

Likewise, other SLRPs via their protein cores can sustain or even enhance receptor signaling, depending on the cellular context. A characteristic example is biglycan, which directly interacts with Insulin-like Growth Factor-I Receptor (IGF-IR), promoting receptor phosphorylation and prolonged activation of downstream PI3K/AKT and ERK pathways, thereby supporting osteosarcoma cell proliferation and resistance to chemotherapy [48]. At the same time, biglycan can function as a danger-associated molecular pattern (DAMP), engaging toll-like receptors 2/4 (TLR2/4) and activating nuclear factor of kappa light-chain enhancer of B cells (NF-κB) signaling, thereby linking ECM remodeling to inflammatory responses [49]. More recent data indicate that biglycan also affects receptor trafficking, including intracellular retention and nuclear translocation of IGF-IR, suggesting that it contributes not only to receptor activation but also to the spatial redistribution and persistence of signaling within the cell [48].

Moreover, protease-dependent SLRP modifications can fine-tune, among others, their receptor- regulating effects [50].

**Figure 1:**
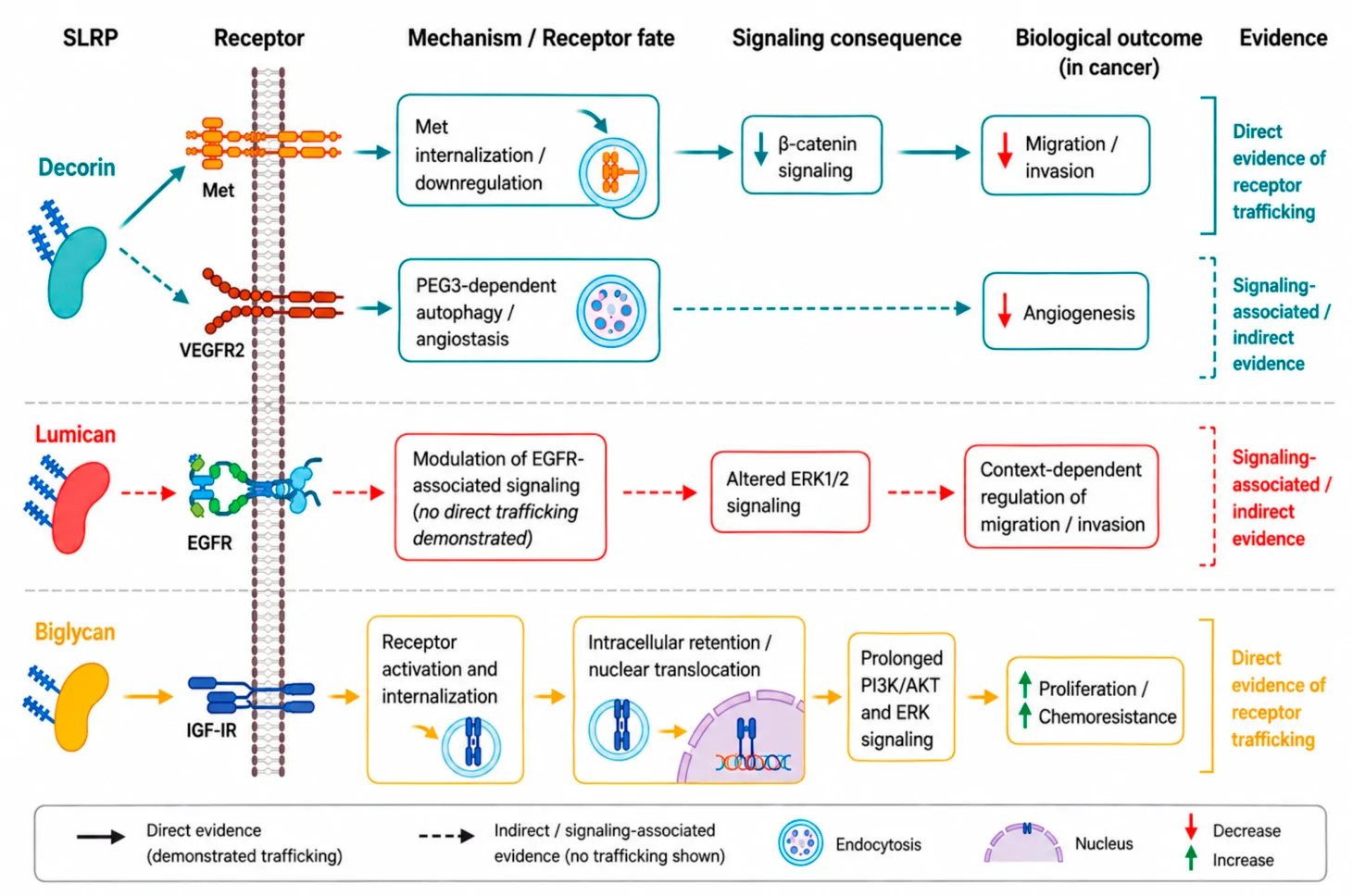
Small leucine-rich proteoglycan (SLRP) core proteins differentially regulate receptor signaling in cancer. Created in BioRender. Nikitovic, D. (2026) https://BioRender.com/wjgumn7. SLRP core proteins interact with or modulate receptor tyrosine kinases (RTKs), thereby affecting receptor activation and downstream signaling and, in selected cases, receptor trafficking. Decorin promotes Met internalization/downregulation, resulting in inhibition of β-catenin downstream signaling and tumor-associated responses. In contrast, lumican modulates EGFR signaling in a context-dependent manner, influencing tumor cell migration and invasion. Biglycan interacts with IGF-IR and promotes sustained receptor activation and intracellular redistribution, including nuclear localization, thereby supporting osteosarcoma cell proliferation and chemoresistance. Together, these examples illustrate that SLRPs can regulate receptor signaling at different spatial levels, although direct effects on receptor fate have been demonstrated only for selected SLRP–receptor pairs.

Indeed, lumican has been shown to modulate growth factor- and integrin-associated signaling pathways, regulating ERK1/2 activity and cytoskeletal organization, ultimately affecting tumor cell migration and invasion [51,52]. In contrast, fibromodulin primarily acts by regulating collagen fibrillogenesis and ECM organization [53], through which it may indirectly influence receptor-associated signaling. These studies, therefore, highlight that SLRP core proteins regulate receptor signaling both directly, through receptor interactions, and indirectly, through remodeling of the extracellular microenvironment (Figure 1).

Importantly, core protein-mediated receptor regulation is not restricted to SLRPs. Transmembrane PGs such as SDCs directly participate in receptor complex assembly, facilitating crosstalk between RTKs and integrins and promoting downstream FAK/Src and MAPK signaling [54,55,56]. Similarly, GPI-anchored glypicans modulate receptor activation, particularly in Wnt signaling, where their core proteins contribute to receptor engagement and signaling specificity [57,58]. Matrix-associated PGs such as perlecan can also directly engage receptors through defined core protein domains, as exemplified by endorepellin-mediated VEGFR2 signaling, which induces receptor internalization and downregulation of the receptor, and angiostatic responses [59,60].

In parallel, GAG chains provide a flexible and highly tunable interface for ligand binding and receptor modulation. Heparan sulfate chains bind numerous growth factors, including fibroblast growth factors (FGFs), vascular endothelial growth factor (VEGF), and hepatocyte growth factor (HGF), thereby stabilizing ligand–receptor interactions, promoting receptor dimerization and regulating local ligand availability within the pericellular microenvironment. Early studies demonstrated that heparan sulfate is essential for FGF–FGFR complex formation and receptor activation [61,62], while structural analyses further revealed that specific sulfation motifs determine ligand binding and receptor specificity [63]. More recent work confirms that alterations in sulfation patterns can bias downstream signaling outputs and contribute to tumor progression [4,40]. Thus, alterations in GAG composition or sulfation may profoundly affect receptor dynamics, signaling microenvironment and cancer-associated signaling reprogramming.

Together, these observations support the concept that core protein-mediated receptor engagement and GAG-dependent ligand organization can cooperate in shaping receptor activation and signaling specificity.

### 2.2. Cell-Surface Organization, Ligand Presentation and Receptor Clustering

The spatial organization of PGs at the cell surface and in the pericellular matrix contributes significantly to the regulation of receptor dynamics and signaling. At the plasma membrane, this organization contributes to receptor clustering, membrane compartmentalization and formation of signaling microdomains [13,14].

Membrane-associated PGs, including SDCs, regulate receptor clustering and the formation of signaling complexes. Pioneer studies demonstrated that SDC-4 cooperates with integrins to regulate focal adhesion formation and signaling [64], while more recent work showed that SDC-2 and SDC-4 form complexes with EGFR and RON kinase, sustaining proliferative signaling in carcinoma cells [65]. These interactions position PGs as organizers of receptor signaling platforms and membrane signaling microdomains [56,66].

At the plasma membrane, PGs further support the interplay between ligands and their receptors by forming ternary signaling complexes that regulate cell growth, migration and adhesion. Acting as co-receptors, PGs enhance the signaling abilities of growth factors by stabilizing ligand–receptor interactions and lowering the threshold required for receptor activation [35]. Importantly, these interactions contribute not only to receptor activation but also to receptor stabilization at the plasma membrane, receptor clustering and spatial restriction of signaling responses within membrane microdomains [6,14]. Furthermore, it is indicated that glycocalyx-associated HSPGs regulate the accessibility and spatial presentation of signaling molecules at the cancer cell surface, emphasizing the importance of PG-rich glycocalyx organization in controlling receptor signaling and membrane receptor accessibility [40]. Beyond this supportive role, PGs can also independently initiate or attenuate signaling cascades related to growth factors and cell-to-cell communication, including synaptogenesis between neurons [34]. Thus, PGs function not only as extracellular binding partners but also as active regulators of membrane signaling organization and signaling configuration [7].

Within the pericellular matrix, PGs regulate ligand distribution and gradient formation. Classic developmental studies demonstrated that heparan sulfate controls morphogen gradients [67], while subsequent work confirmed that HSPGs regulate FGF diffusion and receptor activation in vivo [68]. These principles are now recognized in cancer, where PG-dependent ligand retention contributes to the formation of localized signaling niches that support sustained receptor activation [40,41]. Consequently, PGs contribute to the establishment of spatially restricted signaling environments that regulate receptor accessibility and signaling intensity within tumor-associated microenvironments.

Matrix-associated SLRPs further modulate spatial signaling by altering matrix structure and receptor accessibility. Decorin, for example, regulates collagen organization while simultaneously promoting RTK downregulation [44,45,69]. Thus, PGs integrate biochemical signaling with biomechanical context, influencing receptor accessibility, clustering and signaling output within the tumor microenvironment [4,70].

PGs are also associated with caveolae- and lipid raft-rich membrane domains. Caveolae are cholesterol- and sphingolipid-enriched plasma membrane invaginations that regulate receptor accessibility, membrane organization and mechanosensitive signaling [71]. HSPGs, such as SDCs, localize within these membrane microdomains and participate in receptor clustering and signaling compartmentalization. Lipid raft-associated signaling platforms enriched in PGs regulate receptor accessibility and signaling persistence while simultaneously serving as signaling hubs for RTKs, integrins and GPCRs [13,14]. Thus, PG-associated membrane domains integrate receptor clustering with spatial regulation of signaling responses.

### 2.3. Cancer-Associated PG and GAG Remodeling of Signaling Niches

Cancer-associated changes in PG expression, structure and processing profoundly reshape receptor dynamics and signaling architecture. Altered GAG sulfation patterns, driven by dysregulated sulfotransferases and sulfatases, modify ligand-binding affinities, receptor accessibility and receptor activation thresholds, thereby promoting oncogenic signaling [41]. Because sulfation patterns determine the affinity and selectivity of GAG interactions with growth factors, chemokines and morphogens, even subtle alterations in GAG structure may profoundly affect signaling specificity and the spatial distribution of signaling molecules within the tumor microenvironment. In parallel, upregulation of heparanase enhances growth factor release and signaling, contributing to tumor progression and metastasis [72]. Heparanase-mediated cleavage of heparan sulfate remodels both the glycocalyx and the pericellular ECM, thereby altering receptor accessibility, ligand retention, and stromal communication networks while promoting adaptive signaling within the tumor microenvironment [73,74].

Thus, altered expression of specific PGs further drives receptor-dependent changes in signaling. Increased biglycan expression enhances IGF-IR signaling and promotes proliferation and chemoresistance in osteosarcoma [48], while SDC-1 dysregulation promotes RTK and integrin signaling, enhancing tumor cell migration and invasion. In hepatocellular carcinoma, glypican-3 promotes Wnt/β-catenin signaling by organizing Wnt-associated receptor complexes at the cell surface [57,58], thereby supporting sustained oncogenic signaling and tumor progression [75,76]. Similarly, chondroitin sulfate proteoglycan 4 (CSPG4) enhances integrin-, Src- and FAK-associated signaling pathways in melanoma and triple-negative breast cancer, supporting migratory and invasive tumor phenotypes [77]. These observations highlight that altered PG expression affects tumor progression, actively contributing to the reorganization of signaling and adaptive receptor-associated signaling responses.

Cancer-associated PG remodeling also affects receptor crosstalk and the integration of their respective signaling pathways. Thus, SDCs cooperate with integrins and RTKs within membrane-associated signaling platforms, facilitating coordinated activation of FAK/Src, MAPK and PI3K/AKT pathways [54,56]. Altered PG expression may therefore influence not only individual receptor pathways but also the integration and spatial coordination of multiple signaling cascades within tumor cells.

These molecular alterations are accompanied by remodeling of the pericellular matrix, leading to the formation of aberrant signaling niches where receptor clustering, ligand availability and intracellular trafficking become spatially reprogrammed. Importantly, these signaling niches are highly dynamic microenvironments shaped by continuous ECM remodeling, proteoglycan shedding, altered glycocalyx organization and mechanobiological cues. Consequently, receptor signaling may become spatially restricted, prolonged or redirected toward alternative intracellular pathways associated with survival and therapeutic adaptation.

It is now established that PG-associated alterations in signaling architecture directly contribute to immune modulation within the tumor microenvironment. Specifically, changes in PG and GAG organization regulate cytokine gradients, leukocyte trafficking, and inflammatory signaling pathways, thereby influencing immune cell recruitment and immune-associated signaling responses [78,79]. PG-rich extracellular vesicles also facilitate the dissemination of signaling molecules, proteases, and inflammatory mediators, thereby contributing to stromal activation and premetastatic niche formation [80].

Consequently, PG-dependent signaling reprogramming contributes to phenotypic plasticity, metastatic progression and therapy resistance. Emerging evidence supports a model in which PGs act as central regulators of signaling architecture in cancer. By integrating receptor interactions with GAG-dependent ligand organization, membrane topology and spatial control of receptor trafficking, PGs orchestrate receptor dynamics across cellular compartments, enabling the rewiring of signaling networks that underlie tumor progression and therapeutic resistance.

Together, cancer-associated changes in PG expression, GAG composition and pericellular organization reshape the lateral architecture of receptor signaling at the cell surface and within the tumor microenvironment. These alterations also influence the conditions under which receptors are internalized and subsequently sorted, providing a conceptual link between extracellular signaling organization and the intracellular trafficking mechanisms discussed below.

### 2.4. Receptor Clustering, Co-Receptor Function, Signal Initiation and Signal Strength

Beyond their role in receptor organization at the plasma membrane, PGs also participate in the regulation of receptor trafficking pathways, thereby influencing receptor localization, signaling duration and downstream signaling specificity. Consequently, PGs regulate not only receptor activation at the cell surface, but also the intracellular fate of receptors and the spatial persistence of signaling.

Receptor internalization is a tightly regulated process that controls receptor turnover, recycling and signaling compartmentalization. Internalized receptors may either recycle back to the plasma membrane or undergo lysosomal degradation, with these trafficking decisions profoundly influencing signaling outputs [9,17]. Importantly, receptor signaling is no longer considered restricted to the plasma membrane, as activated receptors may continue signaling within endosomal compartments, thereby contributing to the axial spatial regulation of signaling pathways [7,16].

Clathrin-mediated endocytosis (CME) represents one of the major pathways involved in receptor internalization. During CME, adaptor proteins recognize activated receptors and recruit clathrin-coated pits, ultimately leading to receptor internalization and trafficking into early endosomes [81]. Depending on receptor ubiquitination status and interactions with intracellular sorting machinery, receptors may subsequently recycle to the plasma membrane or be directed toward lysosomal degradation [9]. These trafficking decisions critically influence signaling duration, receptor availability, and receptor resensitization.

Decorin represents one of the best characterized examples of PG-mediated regulation of receptor trafficking. Following binding to EGFR, decorin induces caveolin-1-dependent receptor internalization and subsequent lysosomal degradation, resulting in sustained attenuation of downstream MAPK signaling [45]. Similarly, decorin-mediated Met internalization promotes receptor downregulation and suppression of β-catenin signaling [44]. In endothelial cells, decorin interaction with VEGFR2 activates PEG3-dependent autophagy and angiostatic signaling [46].

In contrast, other PGs appear capable of sustaining receptor signaling through altered receptor trafficking dynamics. Biglycan interaction with IGF-IR promotes prolonged receptor activation and intracellular retention in osteosarcoma cells, while facilitating receptor nuclear translocation [48]. This observation is particularly important because it suggests that PGs may support sustained intracellular signaling rather than simply modulating receptor activation at the plasma membrane. Nuclear localization of RTKs has been associated with transcriptional regulation, tumor progression and therapy resistance [7]. This concept is further supported by recent data on betaglycan/Transforming Growth Factor Beta Receptor 3 (TGFBR3), a membrane proteoglycan that sustains HGF/MET signaling in lung cancer and endothelial cells. In this model, soluble betaglycan enhances HGF-induced MET pathway activation, promoting PI3K/mTOR-associated migratory signaling and tumor growth, thereby illustrating how PGs can modulate receptor signaling persistence and ligand-dependent receptor responsiveness beyond classical Transforming Growth Factor beta (TGF-β) co-receptor activity [82]. These studies establish that proteoglycans do not uniformly regulate receptor trafficking. Whereas decorin primarily directs receptors toward lysosomal degradation and signal termination, biglycan appears to promote receptor retention and nuclear trafficking, illustrating that individual proteoglycans determine distinct intracellular receptor fates and thereby shape the spatial and temporal organization of signaling.

Transmembrane PGs also participate directly in trafficking-associated signaling events. SDCs undergo constitutive endocytosis and recycling, processes that regulate adhesion receptor turnover and focal adhesion dynamics [56]. SDC-4 has been shown to coordinate integrin α5β1 and αVβ3 recycling controlled by SDC-4 phosphorylation, syntenin and Arf6 [83]. Furthermore, SDCs participate in the recycling of adhesion and growth factor receptors by trapping receptors through their glycosaminoglycan chains [84]. Recent work continues to support the role for SDC-4 as a context-dependent regulator of breast cancer cell signaling, adhesion and migration, reinforcing the idea that altered SDC expression can influence receptor-associated signaling routes in tumor cells [85]. Through these interactions, SDCs regulate receptor retention, recycling kinetics and signaling persistence at the plasma membrane.

PGs are also associated with caveolae- and lipid raft-mediated trafficking pathways. Caveolae are flask-shaped plasma membrane invaginations enriched in cholesterol, sphingolipids and caveolins that regulate receptor internalization and mechanosensitive signaling [71]. Cell-surface HSPGs, including SDCs, localize within these membrane microdomains and participate in receptor clustering and signaling compartmentalization. Lipid raft-associated signaling platforms enriched in PGs regulate receptor accessibility and internalization while simultaneously serving as signaling hubs for RTKs, integrins and GPCRs [13,14]. In triple-negative breast cancer cells, experimental tracking of HSPG internalization has further emphasized that cell-surface HSPGs are not static ligand-binding molecules but undergo active endocytosis and intracellular trafficking, processes that may affect cancer cell migration, nutrient handling and signaling compartmentalization [86]. Thus, PG-associated membrane domains integrate receptor clustering with trafficking-dependent regulation of signaling.

Increasing evidence also supports a role for PGs in regulating endosomal signaling platforms. Endosomes are now recognized as active signaling compartments rather than passive transport vesicles [16]. Retention of activated receptors within endosomal compartments may sustain MAPK, PI3K/AKT and Src-family signaling pathways, thereby influencing cell proliferation, migration and survival. Recent mechanobiology studies further support the concept that PG-rich glycocalyx and pericellular matrix organization regulate mechanosensitive receptor function, receptor accessibility, and downstream signaling, thereby linking biochemical receptor regulation with force-dependent signaling and cellular adaptation in cancer-associated microenvironments [4,87]. By modulating receptor internalization, recycling and intracellular retention, PGs may therefore influence not only signaling intensity but also signaling localization and temporal dynamics.

This evidence supports the concept that PGs function as active regulators of receptor trafficking pathways. By modulating receptor internalization, recycling, degradation and intracellular retention, PGs shape the spatial and temporal organization of signaling networks in cancer cells.

### 2.5. Proteoglycan Shedding, GAG and ECM Remodeling, and Extracellular Vesicles

Proteoglycan shedding and ECM remodeling are related but distinct processes that contribute to the dynamic regulation of receptor signaling in cancer.

SDC shedding represents one of the best characterized examples of this process. MMPs and ADAM family proteases cleave SDC ectodomains, generating soluble fragments that retain their capacity to bind growth factors, cytokines and extracellular ligands [88]. Shed SDCs therefore function as soluble signaling platforms that redistribute ligands within the tumor microenvironment and modulate receptor activation at distant cellular sites. Importantly, shedding dynamically alters both the composition of the cell surface glycocalyx and the organization of extracellular signaling niches.

Shed SDC-1 has been associated with enhanced tumor growth, angiogenesis and metastasis in several cancer types, including multiple myeloma and breast carcinoma [89]. In multiple myeloma, shed SDC-1 binds VEGF and promotes angiogenesis by facilitating VEGFR activation on endothelial cells [90]. SDC shedding also promotes integrin and RTK signaling while simultaneously remodeling the pericellular matrix, thereby facilitating tumor cell migration and invasion. Recent data further support that SDC-1 shedding contributes to immune modulation and inflammatory signaling within tumor-associated microenvironments, reinforcing its role as a regulator of both tumor progression and stromal communication [91]. Consequently, proteoglycan shedding contributes to dynamic rewiring of signaling architecture within the tumor microenvironment.

In parallel, enzymatic remodeling of GAG chains represents a distinct process from proteolytic PG shedding. Heparanase cleaves HS chains, enhancing the release of matrix-bound growth factors, including VEGF and FGF, thereby amplifying receptor activation and tumor-associated angiogenesis [72]. Altered sulfation patterns additionally modify ligand-binding affinities and signaling specificity, contributing to adaptive signaling responses in cancer cells [41]. Evidence additionally indicates that dysregulated sulfation patterns influence immune-associated signaling pathways, cytokine gradients and receptor accessibility within tumor-associated extracellular matrices, further contributing to oncogenic signaling adaptation [79]. Thus, both proteolytic processing and structural modification of GAG chains profoundly influence the availability of signaling and receptor activation thresholds.

ECM remodeling additionally alters the biomechanical properties of the tumor microenvironment. Changes in matrix stiffness, collagen organization and glycocalyx composition influence receptor accessibility, integrin activation and mechanotransduction pathways [4,70]. Increased matrix stiffness has been associated with enhanced integrin clustering, focal adhesion formation and sustained RTK signaling in multiple cancer types. More recent mechanobiology-focused studies further indicate that glycocalyx-associated PGs participate in force-dependent receptor organization and mechanosensitive signaling regulation, linking ECM remodeling with altered receptor trafficking and adaptive tumor signaling responses [87]. Accordingly, biochemical and biomechanical remodeling processes cooperate to generate aberrant signaling niches that support tumor progression and therapeutic resistance.

More recently, PGs have also been implicated in extracellular vesicle (EV) biology. PGs contribute to EV biogenesis, cargo organization and intercellular communication, while EV-associated PGs facilitate the dissemination of signaling molecules and matrix-remodeling enzymes within the tumor microenvironment. SDCs and syntenin, for example, participate in exosome biogenesis pathways linked to membrane trafficking machinery [92]. Emerging evidence additionally supports that PG-rich EVs participate in premetastatic niche formation and long-range dissemination of signaling programs by transferring bioactive proteins, cytokines, growth factors and matrix-remodeling enzymes between tumor and stromal cells [80]. These observations further expand the concept that PGs regulate signaling not only locally at the plasma membrane but also across distant cellular and extracellular compartments.

It is now emphasized that ECM dysregulation profoundly alters immune-associated signaling pathways within the tumor microenvironment. Dysregulated ECM components, including PGs and GAG-rich matrices, may function as signaling ligands interacting with immune cell receptors and modulating downstream inflammatory and tumor-promoting signaling cascades [79]. In this context, PGs emerge not only as regulators of receptor signaling in tumor cells, but also as active modulators of stromal and immune-associated communication networks.

Overall, these findings highlight that PG shedding and matrix remodeling are not merely secondary consequences of tumor progression, but active processes that reorganize signaling architecture and receptor dynamics within cancer-associated microenvironments.

## 3. Proteoglycans in Receptor Internalization and Intracellular Trafficking

Beyond their role in organizing receptor signaling at the plasma membrane, PGs may also participate in receptor internalization and intracellular trafficking, thereby influencing receptor localization, signaling duration, and downstream signaling specificity. Receptor internalization constitutes a central component of axial spatial regulation because trafficking decisions determine whether receptors recycle to the plasma membrane, undergo lysosomal degradation, remain within signaling-competent intracellular compartments, or, in selected cases, traffic toward the nucleus [7,9,16,17].

Unlike classical receptor ligands, PGs simultaneously interact with ECM components, soluble ligands, membrane receptors, lipid microdomains, and intracellular trafficking machinery through their modular core proteins and GAG chains. This unique molecular architecture enables PGs to integrate extracellular biochemical and biomechanical information with intracellular receptor trafficking, thereby functioning as continuous organizers of receptor fate rather than isolated signaling modulators. However, the extent of direct evidence for PG-mediated regulation of intracellular trafficking differs considerably among individual PG–receptor systems.

Cell-surface HSPGs, including SDC-1 and SDC-4, function as co-receptors and also regulate cytokinesis and exocytic and endocytic signaling pathways [25,85]. Furthermore, SDC-4 is not only localized to the plasma membrane, but also to endocytic compartments, including early endosomes and multivesicular bodies, indicating active internalization and trafficking along endosomal/lysosomal degradation routes during muscle cell differentiation. Although these findings derive from a non-malignant cellular model, they demonstrate the capacity of SDCs to undergo active intracellular trafficking rather than functioning exclusively as static membrane co-receptors. SDCs are additionally implicated in exosome biogenesis and multivesicular body formation, processes that further connect PGs with intracellular trafficking and intercellular communication [92].

### 3.1. Receptor Internalization and Endocytic-Route Selection

Receptor endocytosis is a fundamental cellular process that regulates signal transduction, receptor availability, and downstream cellular responses. Under physiological conditions, receptor internalization occurs through several well-characterized pathways, with PGs playing important modulatory roles in receptor clustering, ligand presentation, and trafficking dynamics. Importantly, receptor internalization does not simply terminate signaling; it frequently contributes to signal propagation through the spatial redistribution of activated receptors within intracellular compartments.

The route of internalization is influenced by receptor identity, ligand occupancy, membrane organization, and cellular context. Clathrin-mediated endocytosis, caveolae-associated uptake, other clathrin-independent pathways, and macropinocytosis therefore represent mechanistically distinct routes that may result in different intracellular trafficking outcomes.

#### 3.1.1. Clathrin-Mediated Endocytosis

The most extensively studied mechanism is clathrin-mediated endocytosis (CME), which is used by most eukaryotic cells. CME facilitates the uptake of antibodies, hormones, nutrients, and cell-surface receptors, including GPCRs and RTKs, into the cytoplasm. CME is initiated by the recruitment of adaptor protein 2 (AP2) and clathrin triskelions from the cytosol [93]. AP2 acts as the primary orchestrator and is activated through binding to phosphatidylinositol 4,5-bisphosphate (PIP2) and specific cytoplasmic cargo motifs on receptor tails [93]. This recruitment triggers the assembly of a clathrin lattice that deforms the membrane into a clathrin-coated pit (CCP). As the pit matures, GTPase dynamin polymerizes around the neck of the vesicle, facilitating membrane scission via GTP hydrolysis. Finally, auxilin and Hsc70 mediate rapid uncoating of the clathrin lattice, allowing fusion of the vesicle with early endosomes [93]. Although CME represents a major internalization pathway for RTKs and GPCRs, the role of PGs in regulating receptor selection, clustering, and cargo organization during CME remains incompletely understood. During CME, adaptor proteins recognize activated receptors and recruit clathrin-coated pits, ultimately leading to receptor internalization and trafficking into early endosomes [81]. Depending on receptor ubiquitination status and interactions with intracellular sorting machinery, receptors may subsequently recycle to the plasma membrane or be directed toward lysosomal degradation [9]. These trafficking decisions critically influence signaling duration, receptor availability, and receptor resensitization.

Importantly, PG-dependent effects on receptor activation or phosphorylation should not automatically be interpreted as evidence of PG-dependent CME unless receptor internalization or subsequent intracellular sorting has been directly examined.

#### 3.1.2. Caveolae-Associated Endocytosis

Caveolae-associated endocytosis represents a clathrin-independent route operating through cholesterol- and sphingolipid-rich membrane domains enriched in caveolin and cavin proteins. Caveolae are particularly abundant in adipocytes, endothelial cells, fibroblasts, and myocytes. Characterized by 50–80 nm flask-shaped plasma membrane invaginations, these domains are closely associated with the organization of the ECM, glycocalyx, and mechanosensitive signaling [71,94].

This pathway may involve the sequestration and organization of extracellular ligands by HSPGs. Their long, polyanionic GAG chains act as molecular scaffolds capable of binding growth factors, lipoproteins, and pathogens [86,95]. Upon ligand binding, cell-surface HSPGs, including SDCs and glypicans, can undergo lateral diffusion and clustering within cholesterol-enriched lipid rafts. Such clustering contributes to membrane-domain organization and may facilitate association with caveolin- and cavin-containing structures, although the precise relationship between PG clustering and caveolar vesicle formation depends on the cargo and cellular context [86,95]. Thus, cell-surface HSPGs function not only as ligand-binding molecules but also as organizers of membrane microdomains and trafficking platforms.

Caveolin-1 phosphorylation at Tyr14 by Src kinase can contribute to regulation of caveolar internalization. Dynamin subsequently participates in membrane scission, while actin cytoskeleton remodeling supports vesicle internalization [86,95]. Following internalization, caveolae-associated cargo can enter different intracellular routes, including endosomal and transcytotic pathways; receptor fate is therefore not determined solely by caveolar entry. Accordingly, the concept of a discrete “caveosome” is not used here as a general intermediate compartment in caveolar trafficking.

Caveolin- and endocytosis-associated alterations have been investigated in glioma and hepatocellular carcinoma models [96,97]. Given the high hyaluronic acid content of the brain ECM, CD44-mediated interactions have additionally been implicated in glioma cell migration and invasion [96]. These findings illustrate the close relationship between ECM organization, membrane-associated receptors, and invasive signaling, although they do not uniformly provide direct evidence of receptor trafficking.

PGs are also associated with caveolae- and lipid raft-mediated trafficking pathways. Cell-surface HSPGs, including SDCs, localize within these membrane microdomains and participate in receptor clustering and signaling compartmentalization. Lipid raft-associated signaling platforms enriched in PGs regulate receptor accessibility and internalization while simultaneously serving as signaling hubs for RTKs, integrins, and GPCRs [13,14].

In triple-negative breast cancer cells, experimental tracking of HSPG internalization has further emphasized that cell-surface HSPGs are not static ligand-binding molecules but undergo active endocytosis and intracellular trafficking, processes that may affect cancer cell migration, nutrient handling, and signaling compartmentalization [86]. Thus, PG-associated membrane domains integrate receptor clustering with trafficking-dependent regulation of signaling.

#### 3.1.3. Other Clathrin-Independent Routes and Macropinocytosis

Clathrin- and caveolin-independent pathways encompass a heterogeneous group of internalization mechanisms. Among these, the CLIC/GEEC pathway and macropinocytosis represent mechanistically distinct processes and should not be considered as variants of a single endocytic route. The CLIC/GEEC (Clathrin-Independent Carrier/GPI-anchored protein-Enriched Early Endocytic Compartment) pathway is regulated, among other factors, by the GTPase Cdc42 [98].

PGs, particularly cell-surface HSPGs, can participate in clathrin-independent uptake because their polyanionic HS chains bind growth factors, morphogens, and ECM-associated signaling molecules. Acting as co-receptors, SDCs such as SDC-1 interact with SDC-binding protein (SDCBP), linking PG-associated membrane complexes with Rho-family GTPases including RhoA or Arf6 and facilitating clustering of signaling complexes within cholesterol-rich lipid rafts [99,100].

Macropinocytosis, in contrast, involves actin-dependent formation of membrane protrusions and uptake of extracellular fluid and macromolecular cargo. In pancreatic cancer, SDC-1 has been identified as a critical mediator of macropinocytosis, linking PG-dependent membrane organization with nutrient uptake and oncogenic signaling [100].

### 3.2. Endosomal Sorting, Recycling and Degradation

Following internalization, receptor–proteoglycan complexes are sorted within endosomal compartments, where they may be targeted for lysosomal degradation, recycled to the plasma membrane, or continue signaling from endosomes [17,36]. Early endosomes therefore represent major sorting compartments in which receptor fate is determined according to cargo identity, receptor modification, and interactions with intracellular trafficking machinery.

Internalized receptors may recycle back to the plasma membrane, allowing repeated rounds of signaling, or be directed toward lysosomal degradation, resulting in receptor downregulation. These trafficking decisions critically influence signaling duration, receptor availability, and receptor resensitization.

Decorin represents one of the best characterized examples of PG-mediated regulation of receptor trafficking. Following binding to EGFR, decorin induces caveolin-1-dependent receptor internalization and subsequent lysosomal degradation, resulting in sustained attenuation of downstream MAPK signaling [45]. Similarly, decorin-mediated Met internalization promotes receptor downregulation and suppression of β-catenin signaling [44]. In endothelial cells, decorin interaction with VEGFR2 activates PEG3-dependent autophagy and angiostatic signaling [46]. While the EGFR and Met examples directly link decorin to receptor internalization and subsequent receptor fate, the VEGFR2 example primarily demonstrates receptor-associated signaling regulation and should therefore be considered separately when assessing direct trafficking evidence.

Thus, decorin provides a characteristic example in which PG–receptor interaction can promote receptor internalization followed by downregulation and signal termination.

Transmembrane PGs also participate directly in trafficking-associated signaling events. SDCs undergo constitutive endocytosis and recycling, processes that regulate adhesion receptor turnover and focal adhesion dynamics [56]. SDC-4 has been shown to coordinate integrin recycling and directional cell migration through interactions with PKCα and small guanosine triphosphatases (GTPases) [101]. Furthermore, SDCs participate in the recycling of adhesion and growth factor receptors by trapping receptors through their glycosaminoglycan chains [84]. Recent work continues to support a role for SDC-4 as a context-dependent regulator of breast cancer cell signaling, adhesion, and migration, reinforcing the idea that altered SDC expression can influence receptor-associated signaling routes in tumor cells [85]. Through these interactions, SDCs can influence receptor and adhesion-complex recycling, membrane availability, and signaling persistence, although the degree of direct trafficking evidence varies among individual receptor systems.

Recycling is particularly relevant to spatial signaling because return of internalized receptors to the plasma membrane restores receptor availability and can permit repeated rounds of ligand sensing and receptor activation. Conversely, sorting toward late endosomal and lysosomal compartments promotes receptor downregulation and limits signaling persistence. Thus, PG-associated modulation of sorting decisions can potentially shift the balance between receptor resensitization and signal termination.

### 3.3. Endosomal Signaling, Intracellular Retention and Nuclear Trafficking

Increasing evidence supports a role for endosomal compartments as active signaling platforms. Endosomes are now recognized as active signaling compartments rather than passive transport vesicles [16]. Retention of activated receptors within endosomal compartments may sustain MAPK, PI3K/AKT, and Src-family signaling pathways, thereby influencing cell proliferation, migration, and survival.

In some cases, receptors remain within endosomal compartments and continue signaling, a phenomenon referred to as sustained endosomal signaling. The duration and spatial organization of this signaling depend on receptor identity and intracellular sorting, and PG-associated stabilization of receptor–ligand interactions may contribute to these processes in selected systems. However, direct evidence that PGs themselves determine endosomal retention remains limited for several of the examples discussed in this review.

A particularly relevant example is provided by biglycan. Biglycan interaction with IGF-IR promotes prolonged receptor activation and intracellular retention in osteosarcoma cells, while facilitating receptor nuclear translocation [48]. This observation is particularly important because it suggests that PGs may support sustained intracellular signaling rather than simply modulating receptor activation at the plasma membrane. Nuclear localization of RTKs has been associated with transcriptional regulation, tumor progression, and therapy resistance [7].

Biglycan-associated IGF-IR redistribution therefore represents an example in which PG action extends beyond receptor phosphorylation to changes in intracellular receptor localization. These observations distinguish biglycan from PGs such as decorin that predominantly promote receptor downregulation following internalization. Rather than directing receptors toward signal termination, biglycan appears to favor receptor retention within signaling-competent intracellular compartments and facilitate nuclear trafficking of IGF-IR. Although the precise molecular mechanisms remain to be fully elucidated, these findings support the emerging concept that individual PGs differentially regulate receptor fate, thereby determining not only receptor activation but also the spatial compartmentalization and persistence of intracellular signaling [48].

A related but mechanistically distinct example is provided by betaglycan/Transforming Growth Factor Beta Receptor 3 (TGFBR3). Soluble betaglycan enhances HGF-induced MET pathway activation in lung cancer and endothelial cells, promoting PI3K/mTOR-associated migratory signaling and tumor growth, thereby illustrating how PGs can modulate receptor signaling persistence and ligand-dependent receptor responsiveness beyond classical Transforming Growth Factor beta (TGF-β) co-receptor activity [85]. However, these findings demonstrate modulation of MET signaling rather than direct intracellular trafficking of the MET receptor and should therefore not be considered equivalent to the decorin–EGFR/Met or biglycan–IGF-IR trafficking examples.

These studies further illustrate that PGs do not uniformly influence receptor fate. Whereas decorin can direct selected receptors toward internalization, downregulation, and signal termination, biglycan is associated with IGF-IR intracellular retention and nuclear redistribution. The functional outcome of PG–receptor interactions therefore depends on the specific PG, receptor, and cellular context.

Recent mechanobiology studies further support the concept that PG-rich glycocalyx and pericellular matrix organization regulate mechanosensitive receptor function, receptor accessibility, and downstream signaling, thereby linking biochemical receptor regulation with force-dependent signaling and cellular adaptation in cancer-associated microenvironments [87]. These extracellular and membrane-associated effects provide an important connection between lateral spatial organization and receptor behavior, but they should be distinguished from experiments that directly track receptor movement through intracellular compartments.

Together, these observations indicate that PG-associated regulation may be linked to distinct receptor outcomes, ranging from receptor recycling or degradation to sustained intracellular signaling and, in selected receptor systems, nuclear redistribution. Such diversity provides a mechanistic basis for considering receptor fate, rather than receptor phosphorylation alone, as an important dimension of PG-dependent spatial signaling.

Following their organization within cell-surface signaling platforms, receptors may undergo internalization and sorting through distinct intracellular routes. These trafficking decisions determine whether receptors recycle to the plasma membrane, undergo degradation, remain signaling-competent within endosomal compartments, or, in selected cases, traffic to the nucleus. PGs may influence several of these steps through interactions with receptors, membrane microdomains, and trafficking-associated molecules.

**Figure 2:**
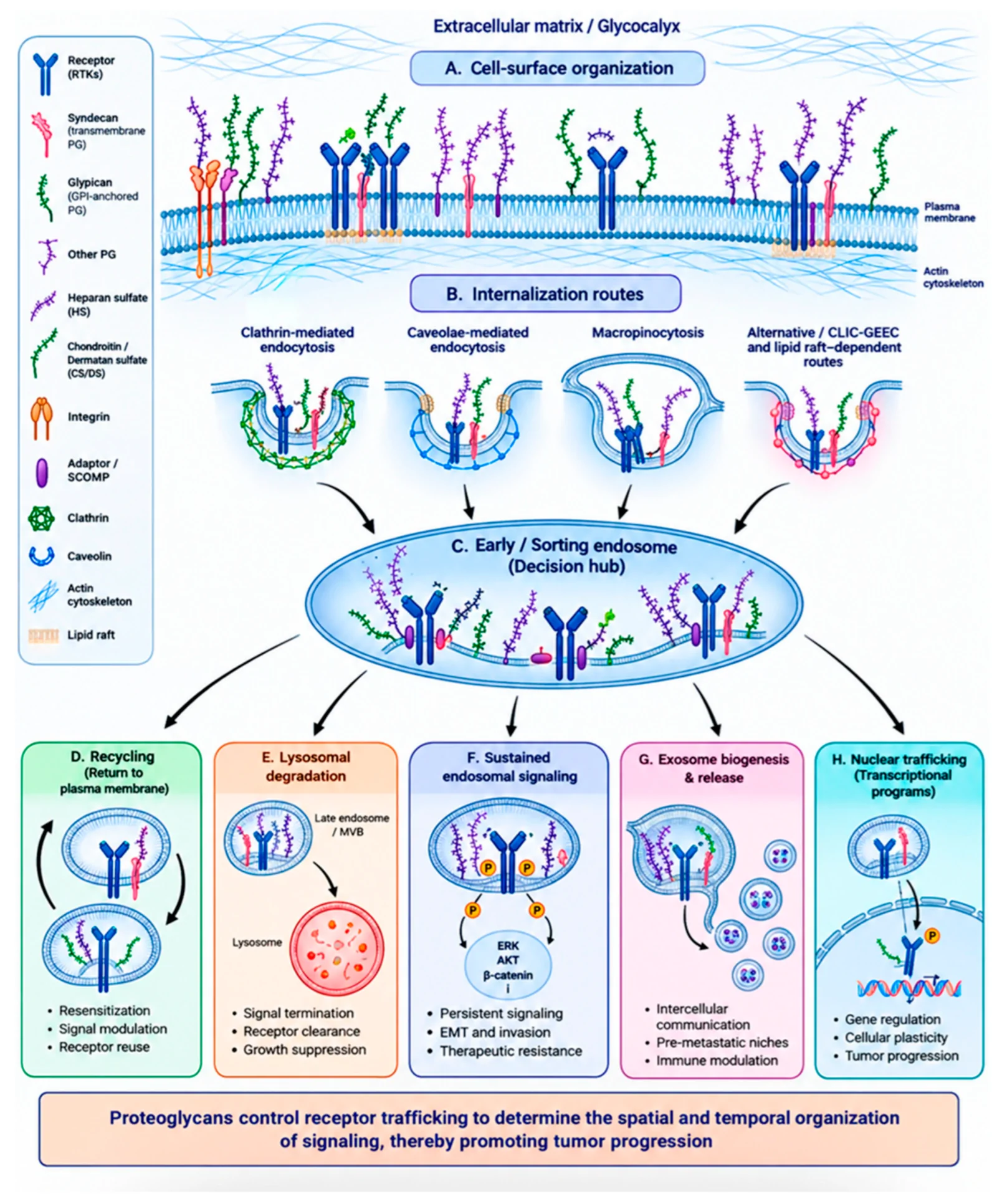
Proteoglycan-dependent regulation of receptor trafficking and spatial signaling. (**A**) Proteoglycans (PGs) contribute to cell-surface organization by modulating ligand presentation, receptor clustering, and interactions with co-receptors and the glycocalyx. (**B**) Receptors may enter clathrin-mediated, caveolae-mediated, macropinocytic, or other clathrin-independent pathways and reach (**C**) early/sorting endosomes. From these compartments, receptors can undergo (**D**) recycling to the plasma membrane, (**E**) trafficking toward late endosomes/lysosomes and degradation, or (**F**) sustained endosomal signaling. (**G**) PGs also participate in multivesicular-body/exosome pathways, contributing to intercellular communication, while (**H**) selected receptors may undergo nuclear trafficking and influence transcriptional programs. The illustrated routes represent possible, context-dependent receptor fates and should not be interpreted as a uniform sequence occurring for all PG–receptor systems. Through these mechanisms, PGs may influence the spatial and temporal organization of receptor signaling, thereby contributing to tumor progression.

Consequently, PGs emerge not only as modulators of receptor activation but also as regulators of intracellular signaling compartmentalization and signaling persistence (Figure 2).

### 3.4. Direct and Indirect Evidence for PG-Associated Receptor Trafficking

The experimental evidence linking individual PGs to receptor trafficking is heterogeneous. Direct evidence is strongest when receptor internalization, intracellular localization, recycling, degradation, endosomal retention, or nuclear redistribution has been experimentally examined. Decorin-mediated EGFR and Met internalization and downregulation, biglycan-associated IGF-IR intracellular redistribution, and selected SDC-dependent recycling events represent examples in which receptor or PG trafficking has been directly addressed.

For other PGs and PG-associated mechanisms, the available studies more commonly demonstrate changes in ligand availability, receptor phosphorylation, downstream signaling, membrane organization, ECM remodeling, or tumor phenotype without directly examining receptor movement between intracellular compartments. Such observations remain relevant to the broader concept of spatial signaling but should be described as indirect or trafficking-associated evidence unless receptor fate has been experimentally demonstrated. This distinction is particularly important for examples involving lumican, fibromodulin, CSPG4, heparanase-associated remodeling, and selected glycocalyx-dependent signaling effects.

The experimental context should also be considered. Some mechanistic observations regarding PG internalization and intracellular localization derive from endothelial, muscle, developmental, or other non-malignant models. These studies provide important mechanistic principles but should be distinguished from trafficking mechanisms demonstrated directly in cancer cells or tumor models.

Accordingly, PGs should not be viewed as uniformly controlling a common receptor-trafficking pathway. Rather, individual PGs regulate receptor organization and signaling at different levels, ranging from ligand presentation and membrane clustering to direct modulation of internalization, recycling, degradation, intracellular retention, or nuclear redistribution. Distinguishing these levels of evidence is essential for defining which PG–receptor interactions represent established trafficking mechanisms, and which remain plausible but incompletely tested components of spatial signaling.

## 4. Proteoglycan Shedding, Extracellular Remodeling and Intercellular Signaling

In addition to regulating receptor organization at the plasma membrane and, in selected systems, intracellular receptor trafficking, PGs undergo extracellular processing that can reshape signaling within the tumor microenvironment. These processes include proteolytic ectodomain shedding, enzymatic remodeling of GAG chains, alterations in ECM and glycocalyx organization, and PG-associated extracellular vesicle signaling. Although interconnected, these mechanisms are biologically distinct and influence receptor signaling through different routes.

### 4.1. Proteolytic Ectodomain Shedding

The functional activity of cell-surface PGs is controlled by a highly regulated mechanism termed ectodomain shedding [88,102,103]. This process involves proteolytic cleavage of the core protein either at the plasma membrane surface or within membrane-associated regions, transforming membrane-bound PGs into soluble bioactive factors [88,102,103]. Importantly, ectodomain shedding reduces the density of membrane-associated PGs while simultaneously remodeling the extracellular signaling environment and altering receptor accessibility, ligand availability, and the spatial distribution of signaling.

Metalloproteinases involved in ectodomain shedding are commonly referred to as sheddases [88,102,103]. Rather than being a simple degradative event, PG shedding can modify cell-surface organization and redistribute PG-bound ligands, thereby influencing the intensity, duration, and spatial localization of receptor-associated signaling.

Several MMPs and ADAM-family proteases mediate cell-surface PG shedding, including that of SDC-1, thereby contributing to dynamic cellular remodeling [88,102,103]. In the case of SDC-1, proteolytic cleavage decreases cell-surface SDC-1 and generates a soluble ectodomain that retains biological activity. Consequently, shedding contributes not only to remodeling of the cell surface and ECM but also to the rewiring of receptor-associated signaling pathways.

When SDC-1 is shed, the resulting soluble ectodomain (sSDC-1) may function either as a carrier or a decoy by sequestering ligands including VEGF and FGF within the ECM, thereby generating potent pro-angiogenic and pro-tumorigenic signaling environments. These soluble ectodomains retain functional GAG chains and therefore preserve the ability to bind growth factors, chemokines, and cytokines, extending signaling regulation beyond the plasma membrane. In parallel, shed SDC ectodomains may also function as dominant-negative regulators by competing with membrane-bound SDCs for ligand binding and growth factor interactions, thereby modulating signaling thresholds within the tumor microenvironment [103,104].

Shed SDC-1 has been associated with enhanced tumor growth, angiogenesis, and metastasis in several cancer types, including multiple myeloma and breast carcinoma [89]. In multiple myeloma, shed SDC-1 binds VEGF and promotes angiogenesis by facilitating VEGFR activation on endothelial cells [90]. SDC shedding also promotes integrin- and RTK-associated signaling while simultaneously remodeling the pericellular matrix, thereby facilitating tumor cell migration and invasion.

Evidence further indicates that shed SDCs contribute to inflammatory signaling and stromal communication within tumor-associated microenvironments, reinforcing their role as active modulators of tumor progression rather than inert degradation products [91]. Indeed, soluble SDC ectodomains have been identified in inflammatory fluids and tissue injury settings, where they regulate growth factor activity, protease function, and leukocyte-associated signaling responses [103].

Heparanase can additionally enhance SDC-1 shedding, thereby linking HS remodeling to ectodomain release without making the two processes mechanistically equivalent. Heparanase-mediated enhancement of SDC-1 shedding promotes aggressive tumor behavior by potentiating VEGF- and HGF-dependent signaling while amplifying inflammatory and angiogenic signaling loops within the tumor microenvironment [74,105].

In malignancies such as endometrial and ovarian cancers, overexpression of sSDC-1 within the stroma promotes release of factors associated with tumor growth and angiogenesis. The prognostic significance of SDC-1 expression appears highly tissue-dependent. In endometrial cancer, altered epithelial and stromal SDC-1 expression has been associated with disease progression and prognosis [106]. These observations highlight that PG shedding may exert context-dependent effects depending on tissue architecture, stromal composition, and receptor-signaling context.

Furthermore, SDC-2 contributes to colorectal cancer progression by regulating MMP-7-associated invasive signaling, while shed SDC-2 can enhance angiogenesis within the tumor microenvironment [107,108]. Similarly, localization of MT1-MMP, CD44, and RhoA within caveolae during endocytosis suggests coordinated interactions between membrane trafficking pathways, cytoskeletal remodeling, and invasive signaling during glioma progression [96]. These observations illustrate crosstalk between extracellular PG processing and membrane-associated signaling rather than demonstrating that shedding itself constitutes an intracellular trafficking mechanism.

Regulation of SDC shedding additionally involves intracellular trafficking-associated molecules. The small GTPase Rab5 directly interacts with the cytoplasmic domain of SDC-1, and dissociation of Rab5 from SDC-1 promotes ectodomain shedding, linking membrane trafficking machinery with extracellular PG processing [88,103]. Moreover, interactions between Rab5 and β1-integrin-associated complexes suggest that integrin signaling and cytoskeletal organization may directly influence shedding dynamics.

Following cleavage, PG ectodomains may function as bioactive effectors or signaling facilitators. Shed SDC-1 and SDC-4 retain their GAG chains and can therefore bind extracellular ligands and modulate their availability and activity [88,103]. In particular, soluble SDC-1 regulates FGF-2 activity and can bind and present angiogenic growth factors, including VEGF, thereby influencing growth factor signaling and angiogenesis [109]. SDC-4 in particular binds CCL19 and facilitates adaptive immunity under physiological conditions. Under pathological conditions, these soluble ectodomains may contribute to tumor invasion, immune modulation, and metastatic dissemination by reshaping extracellular signaling gradients and stromal communication networks.

Clinical prognostic associations, however, represent a distinct level of evidence and should not be inferred directly from these experimental findings. Recent reviews further support that SDC shedding contributes to cancer drug resistance, adaptive stromal signaling, and metastatic dissemination, particularly in tumors characterized by extensive ECM remodeling and chronic inflammatory signaling [110,111].

Shed PG ectodomains may also influence immune-associated signaling through effects on cytokine availability, leukocyte recruitment, and stromal communication. However, proposed effects on immune checkpoint regulation require more direct evidence and are therefore not emphasized here.

**Figure 3:**
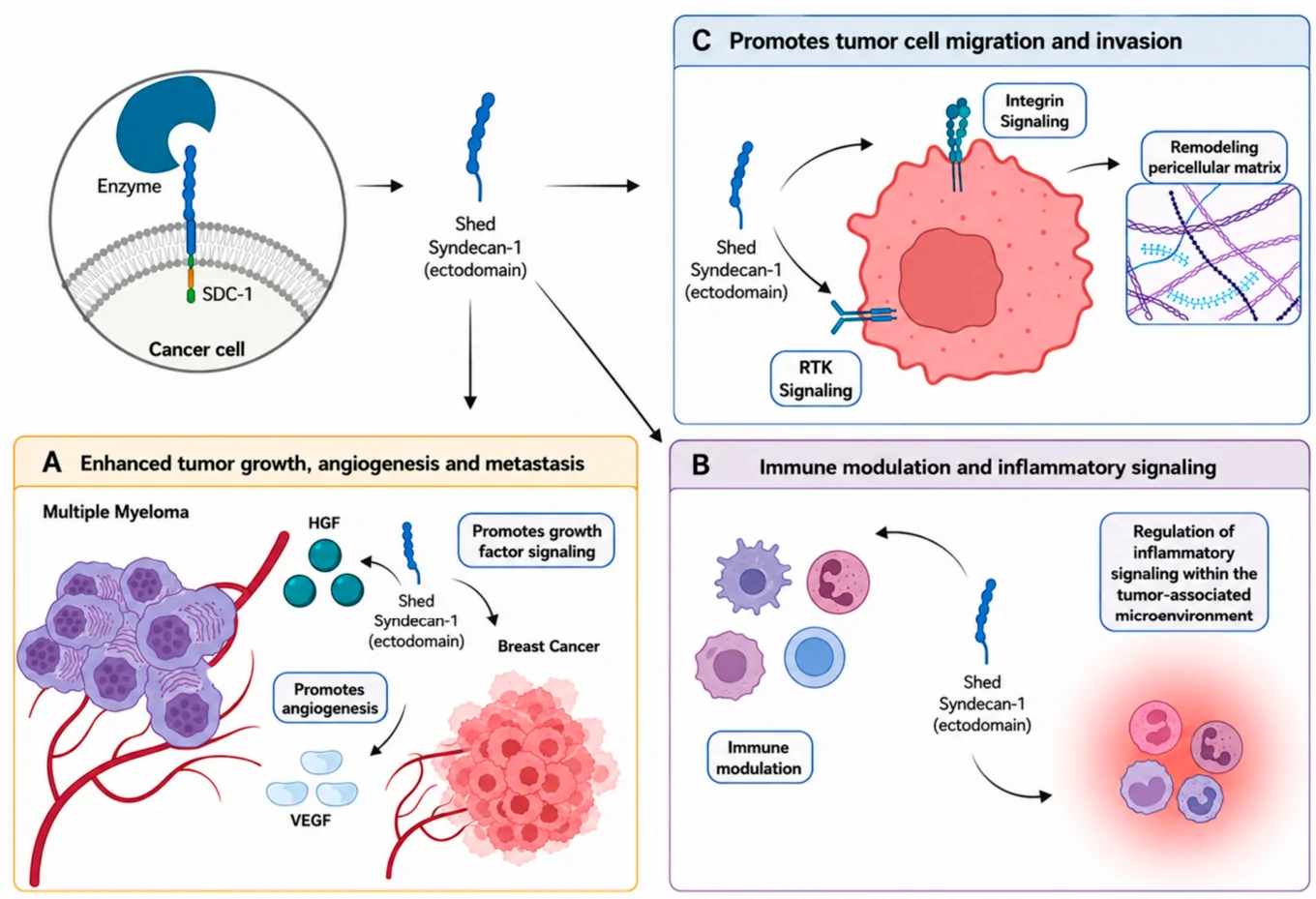
Biological consequences of syndecan-1 shedding in cancer progression. Created in BioRender. Nikitovic, D. (2026) https://BioRender.com/hyy5zw3. Proteolytic cleavage of membrane-bound SDC-1 releases a soluble ectodomain that retains its heparan sulfate chains and associated ligand-binding capacity, thereby extending PG-mediated signaling beyond the cell surface. (**A**) In multiple myeloma and breast cancer, shed SDC-1 binds and redistributes growth factors, including HGF and VEGF, enhancing RTK signaling, angiogenesis, tumor growth, and metastatic dissemination. (**B**) Soluble SDC-1 also contributes to immune modulation by regulating inflammatory signaling and cytokine activity within the tumor-associated microenvironment, thereby influencing immune cell recruitment and stromal communication. (**C**) In solid tumors, shed SDC-1 promotes cancer cell migration and invasion by facilitating RTK- and integrin-associated signaling while contributing to remodeling of the pericellular ECM.

Different biological outputs of SDC-1 shedding are illustrated in Figure 3.

Thus, SDC-1 shedding converts a membrane-associated PG into a soluble regulator of extracellular signaling, with consequences for growth-factor distribution, angiogenesis, stromal communication, and invasion.

### 4.2. GAG Remodeling and Heparanase

Proteolytic cleavage of PG core proteins should be distinguished from enzymatic remodeling of their GAG chains. Whereas ectodomain shedding releases part of the PG core protein together with its associated GAG chains, heparanase acts primarily by cleaving HS chains and thereby modifying the extracellular distribution and availability of HS-bound signaling molecules.

In parallel with PG shedding, enzymatic remodeling of GAG chains modifies signaling niche organization. Heparanase-mediated cleavage of heparan sulfate enhances the release of matrix-bound growth factors, including VEGF and FGF, thereby amplifying receptor activation and tumor-associated angiogenesis [72].

Heparanase-mediated HS cleavage also remodels the glycocalyx and pericellular matrix, altering ligand retention, receptor accessibility, and stromal communication. These effects can indirectly reshape receptor-associated signaling without necessarily involving direct regulation of receptor intracellular trafficking.

Altered sulfation patterns additionally modify ligand-binding affinities and signaling specificity, contributing to adaptive signaling responses in cancer cells [41]. Evidence additionally indicates that dysregulated sulfation patterns influence immune-associated signaling pathways, cytokine gradients, and receptor accessibility within tumor-associated extracellular matrices, further contributing to oncogenic signaling adaptation [79]. Thus, both enzymatic cleavage and structural modification of GAG chains can profoundly influence ligand availability and receptor activation thresholds. The original Section 2.5 already developed these GAG-dependent effects separately from the SDC examples.

Interestingly, HS chains themselves may regulate susceptibility to shedding. Experimental studies have demonstrated that intact HS chains suppress SDC-1 shedding, whereas alterations or reductions in HS chains enhance ectodomain release, emphasizing the importance of GAG structural integrity in regulating PG processing [104].

This observation provides a mechanistic link between GAG remodeling and ectodomain shedding while preserving the distinction between cleavage of HS chains and proteolytic cleavage of the PG core protein.

### 4.3. ECM and Glycocalyx Remodeling

ECM remodeling additionally alters the biomechanical properties of the tumor microenvironment. Changes in matrix stiffness, collagen organization, and glycocalyx composition influence receptor accessibility, integrin activation, and mechanotransduction pathways [4,70]. Increased matrix stiffness has been associated with enhanced integrin clustering, focal adhesion formation, and sustained RTK signaling in multiple cancer types.

More recent mechanobiology-focused studies further indicate that glycocalyx-associated PGs participate in force-dependent receptor organization and mechanosensitive signaling regulation, linking ECM remodeling with adaptive receptor-associated signaling [87]. Accordingly, biochemical and biomechanical remodeling processes cooperate to generate aberrant signaling niches that support tumor progression and therapeutic resistance.

These effects are conceptually distinct from receptor trafficking. ECM stiffness, glycocalyx architecture, and pericellular matrix composition primarily modify receptor accessibility, clustering, adhesion, and mechanotransduction; they may consequently influence receptor behavior, but should not be described as direct trafficking mechanisms unless intracellular receptor movement has been experimentally demonstrated.

### 4.4. Extracellular Vesicles and Long-Range Signaling

More recently, PGs have also been implicated in extracellular vesicle (EV) biology. PGs contribute to EV biogenesis, cargo organization, and intercellular communication, while EV-associated PGs facilitate the dissemination of signaling molecules and matrix-remodeling enzymes within the tumor microenvironment.

SDCs and syntenin, for example, participate in exosome biogenesis pathways linked to membrane trafficking machinery [92]. Exosome formation occurs through late endosomal/multivesicular-body pathways, providing a connection between intracellular membrane trafficking and extracellular release without implying direct formation of exosomes from early endosomes.

Emerging evidence additionally supports that PG-rich EVs participate in premetastatic niche formation and long-range dissemination of signaling programs by transferring bioactive proteins, cytokines, growth factors, and matrix-remodeling enzymes between tumor and stromal cells [80]. These observations further expand the concept that PGs regulate signaling not only locally at the plasma membrane but also across distant cellular and extracellular compartments.

Shed PG ectodomains and EV-associated PGs therefore represent two distinct mechanisms by which PG-dependent signaling can extend beyond the cell of origin: soluble ectodomains redistribute extracellular ligands, whereas EVs transport membrane-associated and intravesicular signaling cargo between cells.

Overall, PG shedding, GAG remodeling, ECM/glycocalyx reorganization, and EV-associated communication constitute complementary but mechanistically distinct mechanisms for remodeling signaling within the tumor microenvironment. Together, they connect local changes in cell-surface organization with broader stromal, angiogenic, inflammatory, and intercellular signaling networks that contribute to cancer progression.

## 5. Therapeutic Implications

The therapeutic relevance of PGs derives from several complementary properties, including tumor-associated expression, cell-surface accessibility, regulation of growth-factor and receptor signaling, and, for selected targets, efficient internalization following ligand or antibody binding. The examples discussed below—SDC-1/CD138, GPC3, CSPG4 and heparanase—illustrate distinct strategies for targeting PG-dependent signaling or exploiting PGs as tumor-associated surface molecules. Importantly, receptor or target internalization is mechanistically relevant for some therapeutic modalities, particularly ADCs and immunotoxins, but is not required for all PG-directed approaches.

### 5.1. CD138-Based Strategies (CD138)

CD138, also known as SDC-1, is a transmembrane HSPG with a major role in the progression of several cancer types, particularly Multiple Myeloma (MM). SDC-1 is highly expressed in malignant plasma cells and participates in tumor growth, survival and proliferation through binding of growth factors and ECM components. In parallel, dysregulated shedding of SDC-1 contributes to angiogenesis, stromal remodeling and tumor dissemination, making it an attractive therapeutic target for immune-based strategies [112]. Beyond functioning as the clinically established CD138 plasma-cell marker, SDC-1 is increasingly recognized as an active regulator of receptor signaling, growth factor retention and tumor–stroma communication within the bone marrow microenvironment.

MM is a hematological malignancy caused by malignant plasma cells localized primarily in the bone marrow. The affected plasma cells proliferate uncontrollably and overproduce monoclonal immunoglobulins, leading to organ damage [113]. MM is the second most common hematological malignancy [114]. The role of SDC-1 in MM progression is well established, with elevated SDC-1 expression correlating with disease severity and progression [115]. Increased SDC-1 shedding, closely associated with heparanase activity, contributes to MM progression and remodeling of the bone marrow microenvironment [90,105,116,117]. Notably, the heparanase/SDC-1 axis promotes growth factor release, receptor activation and inflammatory signaling within the MM microenvironment, thereby facilitating angiogenesis, metastatic dissemination and therapy resistance [74,105].

SDC-1 additionally promotes angiogenesis in multiple myeloma through enhancement of VEGF–VEGFR2 signaling in endothelial cells [90]. More recent data suggest that shed SDC-1 functions as a soluble signaling platform that concentrates and redistributes heparan sulfate-binding growth factors, including VEGF, HGF, and FGF family members, within the bone marrow niche, thereby sustaining RTK signaling and adaptive tumor–stromal communication [112,117]. The use of CD138 as a therapeutic target in MM therefore represents a promising strategy and remains the focus of extensive investigation. Its accessible membrane localization and central role in malignant plasma cell biology make SDC-1 particularly attractive for the treatment of aggressive and relapsed disease. Monoclonal antibodies [118], antibody–drug conjugates (ADCs) [112], radioimmunoconjugates [119] and immunocytokines [120] have all been developed to selectively eliminate MM cells through CD138 targeting. For payload-delivery strategies such as ADCs, target internalization and subsequent intracellular routing are important determinants of therapeutic efficacy because the conjugated cytotoxic agent must reach the appropriate intracellular compartment. In contrast, therapeutic antibodies, radioimmunoconjugates and immune-recruiting approaches may depend primarily on target accessibility rather than intracellular trafficking.

More recently, next-generation immunotherapeutic approaches combining CD138 targeting with immune modulation, cytokine delivery or engineered cellular therapies have shown increasing promise in preclinical and early translational studies, particularly in treatment-resistant MM subtypes [112]. However, many of these approaches remain associated with significant cytotoxicity-related adverse effects.

A further consideration is SDC-1 shedding. Soluble CD138 may sequester CD138-directed agents or otherwise alter their distribution within the tumor microenvironment, potentially affecting the efficacy of therapies that rely on recognition of membrane-associated CD138. This possibility warrants consideration, although its quantitative clinical relevance varies among individual therapeutic platforms.

### 5.2. GPC3-Based Strategies

Glypican-3 (GPC3), is a membrane-associated heparan sulfate proteoglycan with a major role in signaling pathways involved in cancer progression, particularly hepatocellular carcinoma (HCC). GPC3 expression is low or absent in normal liver tissue but strongly elevated at the surface of liver cancer cells. This proteoglycan promotes tumor growth, metastasis and resistance to therapy, while its selective tumor-associated expression makes it an attractive tumor-specific antigen for targeted therapies [121]. Importantly, GPC3 functions not merely as a biomarker but as an active organizer of oncogenic signaling complexes regulating Wnt, Hedgehog, Hippo/YAP and growth factor-associated pathways. Advanced HCC frequently requires systemic therapy, but treatment efficacy remains limited in a substantial proportion of patients. The selective expression of GPC3 on HCC cells therefore provides an attractive opportunity for tumor-directed therapeutic approaches [121,122].

Because the treatment landscape of advanced HCC is rapidly evolving, the relevant point for the present review is that advanced disease frequently requires systemic therapy and that tumor-associated surface molecules such as GPC3 provide opportunities for more selective therapeutic targeting [122]. Early-stage disease may be treated surgically with relatively favorable survival rates; however, asymptomatic progression frequently delays diagnosis. Advanced HCC generally requires systemic treatment, with current therapeutic approaches increasingly centered on immunotherapy-based regimens, alongside molecularly targeted therapies [121].

GPC3 is a central regulator of multiple signaling pathways necessary for HCC progression. Within canonical Wnt/β-catenin signaling, GPC3 functions as a co-receptor facilitating β-catenin nuclear translocation and activation of oncogenic targets including c-Myc and Cyclin D1, thereby promoting proliferation, resistance to apoptosis and aggressive tumor behavior [75,76]. GPC3 also interacts with the Hippo/YAP signaling pathway, and GPC3 knockdown in Huh7 cells reduces YAP expression and inhibits proliferation [123]. GPC3 also promotes the assembly of Wnt–Frizzled signaling complexes at the cell surface through direct interaction with Frizzled, supporting its role in the spatial organization of canonical Wnt signaling [58]. GPC3 also promotes epithelial–mesenchymal transition (EMT) in HCC cells through activation of ERK signaling, thereby enhancing cell migration and invasion [124]; and enhances angiogenesis through interactions associated with Hedgehog signaling pathways [125]. Current and emerging GPC3-based therapies represent a promising therapeutic avenue. These approaches include monoclonal antibodies, bispecific antibodies, Chimeric Antigen Receptor T-cell (CAR-T) cell therapies and immunotoxins. One of the best characterized anti-GPC3 antibodies is Codrituzumab, although its clinical development was limited by insufficient efficacy [126]. This negative result is informative because tumor-selective antigen expression alone does not guarantee therapeutic efficacy; target density, accessibility, tumor heterogeneity, immune effector engagement, and the biological consequences of target binding can all influence treatment outcome. More recently, bispecific antibodies targeting both GPC3 and CD16A have been developed in order to enhance antibody-dependent cellular cytotoxicity (ADCC) through recruitment of NK cells, thereby improving tumor-selective immune responses [127]. These approaches are particularly attractive because they combine tumor-specific targeting with modulation of immune-associated receptor signaling pathways.

CAR-T cell therapy represents another major strategy. GPC3-directed CAR-T therapy has progressed beyond purely preclinical evaluation, with early clinical studies demonstrating the feasibility of this strategy [128]. More recent efforts additionally focus on improving CAR-T persistence, trafficking and local tumor infiltration within the highly immunosuppressive HCC microenvironment.

Immunotoxins targeting GPC3 also demonstrate promising anti-tumor activity. Cytotoxic agents coupled to GPC3-targeting antibodies inhibit Wnt signaling and protein synthesis, thereby promoting tumor regression [129]. For such payload-delivery approaches, internalization and appropriate intracellular routing are required for cytotoxic activity.

### 5.3. CSPG4-Based Strategies

CSPG4 is a transmembrane glycoprotein/proteoglycan overexpressed in several malignancies, including melanoma, triple-negative breast cancer, rhabdomyosarcoma, and acute lymphoblastic leukemia. Its central role in tumor-promoting signaling, together with its tumor-selective overexpression, makes CSPG4 an attractive therapeutic target [77]. Beyond serving as a tumor-associated antigen, CSPG4 actively regulates receptor-associated signaling pathways controlling migration, survival, invasion and ECM interactions.

CSPG4 participates extensively in oncogenic signal transduction. Its extracellular domain binds growth factors and ECM-associated ligands, thereby activating mitogenic signaling pathways and transcriptional programs [130]. Downstream, the intracellular domain promotes survival signaling, proliferation, EMT and tumor cell motility [77,131]. A characteristic example is the interaction of CSPG4 with ECM components and integrins. CSPG4 acts as a co-receptor promoting integrin and fibronectin clustering, which subsequently induces the formation of filopodia and migratory cellular extensions necessary for invasion [132]. CSPG4-associated chondroitin sulfate also contributes to pro-MMP-2 activation, providing an additional mechanism linking CSPG4 to matrix remodeling and melanoma invasion [133]. These studies primarily demonstrate CSPG4-dependent signaling and membrane organization rather than direct receptor trafficking, consistent with its classification in Table 1 as indirect trafficking evidence.

As expected from its signaling functions, CSPG4 represents a promising target for multiple tumor-selective therapeutic approaches, including monoclonal antibodies, ADCs, immunotoxins and TRAIL fusion proteins [77]. Several monoclonal antibodies successfully inhibited the growth and progression of CSPG4-positive tumors. For example, mAb 9.2.27 effectively inhibited melanoma progression, likely through blockade of ECM ligand interactions with the CSPG4 extracellular domain [134]. Similarly, mAb 225.28 suppressed proliferation and metastasis in breast cancer by inhibiting CSPG4-mediated survival signaling [135].

Targeted nanoparticle-based delivery systems have also been developed. HFt-Pt-Ep1, combining CSPG4 recognition with cisplatin-loaded human ferritin nanoparticles, demonstrated selective anti-tumor activity against CSPG4-positive cancers with limited toxicity toward CSPG4-negative cells [136]. Notably, surface accessibility alone supports selective targeting, whereas efficient payload delivery additionally requires internalization and intracellular processing.

Protein-based immunotoxins additionally represent promising therapeutic alternatives. Brehm et al. demonstrated that a truncated Exotoxin A (ETA’) targeting CSPG4 induced rapid internalization and apoptosis in tumor cells [137]. This represents a therapeutically relevant trafficking event because internalization permits intracellular delivery of the cytotoxic payload.

Another attractive strategy for aggressive tumors involves activation of death receptors through TRAIL-associated therapies. TRAIL fusion proteins utilize tumor-associated antigens such as CSPG4 to selectively activate apoptotic pathways in cancer cells. CSPG4:sTRAIL specifically targets melanoma cells characterized by high CSPG4 expression, inducing apoptosis with substantial specificity [77,138]. Thus, CSPG4-directed therapies exploit different aspects of CSPG4 biology: some depend primarily on surface recognition or blockade of signaling complexes, whereas immunotoxin and payload-delivery approaches additionally benefit from target internalization. As for SDC-1, soluble or shed CSPG4 may theoretically influence the distribution of CSPG4-directed antibodies or payload-bearing agents. This possibility is relevant to therapeutic design but should not be overstated in the absence of direct evidence for individual agents.

### 5.4. Heparanase-Based Strategies

Heparanase is a heparan sulfate-degrading endo-β-D-glucuronidase with major involvement in tumor invasion, angiogenesis and metastasis. Overexpressed in numerous human malignancies, heparanase regulates ECM remodeling, growth factor release and tumor-associated signaling pathways, making it a promising therapeutic target [139]. Current therapeutic approaches targeting heparanase include heparin analogues, polysulfated saccharides, nucleic acid-based inhibitors and therapeutic antibodies [140]. Importantly, heparanase is now recognized not only as an ECM-degrading enzyme, but also as a regulator of receptor signaling availability, SDC shedding and tumor-associated signaling niche remodeling.

Heparan sulfate chains are abundant in ECM PGs and basement membranes. Consequently, heparanase-mediated cleavage disrupts ECM barriers while simultaneously releasing matrix-bound growth factors and cytokines. These processes directly promote tumor invasion, angiogenesis and metastasis [140,141]. Through release of VEGF, FGF and HGF family members, heparanase profoundly alters RTK-associated signaling pathways within tumor-associated microenvironments.

Furthermore, increasing evidence supports a direct heparanase/SDC-1 signaling axis. Within the tumor microenvironment, heparanase enhances SDC-1 shedding, thereby altering tumor–stromal communication and promoting migration, angiogenesis and metastasis [142]. Recent studies additionally indicate that the heparanase/SDCs axis contributes to inflammatory signaling, immune modulation and adaptive resistance pathways in multiple malignancies [74,105]. These effects primarily concern extracellular remodeling, ligand availability, and signaling-niche organization; they should not be presented as direct receptor-trafficking mechanisms.

Several classes of heparanase inhibitors have therefore been developed. Heparin and heparin analogs represent potent inhibitors, although the clinical use of native heparin is limited by bleeding complications. Low-molecular-weight heparins, including dalteparin, tinzaparin and enoxaparin, should not be considered simple heparanase-specific anticancer drugs. Their biological effects include anticoagulant and other pleiotropic activities, and any contribution of direct heparanase inhibition should therefore be distinguished from these broader actions [143,144]. Polysulfated saccharides additionally represent an important class of experimental anti-cancer agents. These compounds mimic natural heparan sulfate and bind heparanase, HS-binding growth factors, chemokines and ECM proteins [145]. Several of these compounds demonstrated substantial anti-tumor activity [140].

Nucleic acid-based inhibitors represent another major therapeutic category. These approaches include antisense oligonucleotides, ribozymes, aptamers, siRNA, shRNA and miRNA-based therapeutics specifically targeting heparanase expression. Many demonstrated significant anti-tumor effects in preclinical studies [140]. More recent RNA-based therapeutic approaches additionally focus on selective modulation of tumor-associated signaling networks regulated by heparanase rather than simple enzymatic inhibition alone.

Therapeutic antibodies additionally represent a promising anti-heparanase strategy. Antibodies generated against selected human heparanase epitopes reduced heparanase-associated invasive activity in HCC models [146]. Heparanase-neutralizing monoclonal antibodies inhibited tumor growth and metastatic dissemination in lymphoma models [147]. Unlike ADC- or immunotoxin-based targeting of membrane PGs, heparanase inhibition does not principally depend on receptor internalization. Its therapeutic rationale is instead based on restricting HS cleavage, growth-factor mobilization, SDC shedding, and remodeling of tumor-associated signaling niches.

Together, these examples illustrate that therapeutic targeting of PG biology can operate at different spatial levels: direct targeting of membrane PGs, exploitation of target internalization for intracellular payload delivery, interference with extracellular ligand organization, or inhibition of enzymatic ECM/GAG remodeling. Representative PG-directed therapeutic strategies, their clinical context, and their relationship to target internalization or extracellular signaling regulation are summarized in Table 2.

## 6. Conclusions

Proteoglycans are increasingly recognized as active regulators of the spatial organization of receptor signaling rather than solely as structural components of the ECM or passive co-receptors. Through their core proteins and GAG chains, PGs influence ligand presentation, receptor accessibility and clustering, membrane-domain organization, and the composition of pericellular signaling niches [1,4,6,14]. Cancer-associated changes in PG expression, GAG composition and sulfation, glycocalyx organization, and ECM remodeling can therefore reshape receptor signaling at multiple spatial levels and contribute to tumor-cell plasticity, invasion, and therapeutic resistance [4,40,41].

An important conclusion emerging from the available evidence, however, is that PG-dependent regulation of receptor signaling should be distinguished from direct regulation of receptor trafficking. For selected PG–receptor systems, trafficking has been demonstrated experimentally. Decorin promotes internalization and downregulation of EGFR and Met [44,45], whereas biglycan is associated with intracellular retention and nuclear redistribution of IGF-IR [48], and SDC-dependent mechanisms can regulate recycling of adhesion receptors [101]. In contrast, for several other PGs, including lumican, fibromodulin and CSPG4, current evidence primarily demonstrates effects on ligand availability, receptor activation, downstream signaling, membrane organization, or ECM remodeling rather than direct receptor trafficking [51,52,53,77]. This distinction is important for defining the mechanistic boundaries of the emerging concept of PG-dependent spatial signaling. PG processing further extends these regulatory functions beyond the plasma membrane. Proteolytic shedding generates soluble PG ectodomains capable of redistributing growth factors and cytokines [88,89,102,103], whereas heparanase-mediated HS cleavage and changes in GAG sulfation remodel ligand availability and signaling gradients [41,72,73,74]. PG-associated extracellular vesicles provide an additional mechanism for long-range intercellular communication through the syndecan–syntenin–ALIX pathway [80,92]. These processes illustrate that PG-dependent spatial signaling encompasses not only intracellular receptor fate but also dynamic communication between the cell surface, ECM, glycocalyx, stromal compartments, and distant recipient cells. Therapeutically, this complexity creates distinct opportunities: membrane PGs can serve as tumor-associated targets for antibodies, cellular therapies and payload-delivery strategies, while extracellular PG/GAG remodeling can be targeted to disrupt tumor-supportive signaling niches [112,126,127,128,129,130,131,132,133,134,135,136,137,138,139,140,141,142,143,144,145,146,147].

Future studies should define these mechanisms with greater spatial and temporal resolution. Live-cell imaging, receptor-specific internalization and recycling assays, quantitative endosomal colocalization, spatial proteomics, and approaches that distinguish plasma-membrane, endosomal, lysosomal, and nuclear receptor pools will be particularly valuable. Equally important will be validation in three-dimensional tumor models, organoids, patient-derived samples, and spatially resolved tissue systems that preserve the complex relationship between tumor cells, stromal cells, the glycocalyx, and the ECM. Such approaches should help establish which PG–receptor interactions directly determine receptor fate, which primarily regulate the extracellular signaling environment, and how these mechanisms can be exploited therapeutically. A more precise understanding of these context-dependent functions may ultimately enable PG-associated spatial signaling to be translated into more selective strategies for targeting cancer progression and therapeutic resistance.

## Figures and Tables

**Table 1:** Comparison between the role of proteoglycans and GAGs in endocytic pathways and cancers.

Proteoglycan/PG System	Receptor/Partner	Cancer Type/Experimental Model	Experimental Approach/Evidence	Trafficking Step and Receptor Fate	Functional Consequence	Evidence Level
Decorin	EGFR	A431 squamous carcinoma and other transformed cells	Receptor internalization and degradation were directly examined; caveolin-dependent uptake was implicated [45]	Caveolae-associated internalization → lysosomal degradation	Sustained attenuation of EGFR/MAPK signaling and inhibition of proliferation	Direct
Decorin	Met	Tumor cell models	Receptor internalization/downregulation and intracellular degradation were examined [44]	Internalization → receptor downregulation/degradation	Reduced β-catenin signaling, migration and invasion	Direct
Decorin	VEGFR2	Endothelial cells	VEGFR2-associated signaling and PEG3-dependent autophagy were examined [46]	Receptor fate not directly established	Autophagy and angiostatic signaling	Indirect/signaling-associated
Biglycan	IGF-IR	Osteosarcoma cells	Intracellular receptor redistribution and nuclear localization were examined in addition to receptor activation [48]	Internalization/intracellular retention → nuclear translocation	Prolonged IGF-IR signaling, proliferation and chemoresistance	Direct
SDC-4	Integrins	Cell migration models	Integrin recycling and directional migration were examined [101]	Integrin recycling → plasma-membrane redistribution	Focal-adhesion dynamics and directional migration	Direct
Cell-surface HSPGs	HSPG-associated cargo	Triple-negative breast cancer cells	Experimental tracking demonstrated HSPG internalization and intracellular trafficking [86]	PG endocytosis → intracellular redistribution	Associated with migration, nutrient handling and signaling compartmentalization	Direct for PG trafficking; indirect for receptor fate
Betaglycan/TGFBR3	MET	Lung cancer and endothelial models	HGF-dependent MET activation and downstream signaling were examined [82]	Specific receptor-trafficking step not demonstrated	PI3K/mTOR-associated migratory signaling and tumor growth	Indirect
Lumican	EGFR/integrin-associated pathways	Melanoma and other tumor models	Receptor/downstream signaling, cytoskeletal organization and migration were examined [51,52]	Receptor trafficking not directly demonstrated	Context-dependent regulation of migration and invasion	Indirect
Fibromodulin	Integrin-associated signaling	ECM/cell-adhesion models	ECM organization and FAK/Src-associated signaling were examined [53]	Receptor trafficking not directly demonstrated	Modulation of adhesion-associated signaling	Indirect
CSPG4	Integrin/Src/FAK-associated complexes	Melanoma, TNBC and other cancers	Receptor-associated signaling and tumor-cell phenotype were examined [77]	Receptor trafficking not directly demonstrated	Enhanced proliferative, migratory and invasive signaling	Indirect
PG-rich glycocalyx	Mechanosensitive receptors/integrins	Cancer-associated mechanobiology models	Glycocalyx organization, receptor accessibility and mechanosensitive signaling were examined [87]	Intracellular receptor fate not directly examined	Force-dependent signaling and cellular adaptation	Indirect/spatial signaling

**Table 2:** Therapeutic targeting strategies involving proteoglycans and proteoglycan-associated molecules in cancer.

Target	Cancer Type/Clinical Context	Biological/Therapeutic Rationale	Representative Therapeutic Strategies	Translational Status/Key Consideration
SDC-1 (CD138)	Multiple myeloma; particularly relapsed/refractory disease	Highly expressed on malignant plasma cells; regulates growth-factor signaling, tumor–stroma interactions and angiogenesis; increased shed SDC-1 is associated with disease progression [112,115,116,117]	Monoclonal antibodies, ADCs, radioimmunoconjugates and immunocytokines [112,118,119,120]	CD138-directed approaches include preclinical and clinical-stage strategies. Internalization is particularly relevant for ADC-mediated payload delivery, whereas SDC-1 shedding may influence accessibility of membrane CD138.
GPC3	Hepatocellular carcinoma; particularly advanced/unresectable GPC3-positive disease	Tumor-associated surface expression; contributes to Wnt and other oncogenic signaling pathways [57,58,75,76,121,123,124,125]	Codrituzumab, bispecific antibodies, GPC3-directed CAR-T cells and immunotoxins [126,127,128]	Codrituzumab showed insufficient efficacy in a randomized Phase II study [126], whereas GPC3 CAR-T therapy has reached early clinical evaluation [128]. Internalization is relevant for immunotoxin/payload-delivery approaches but not required for CAR-T-cell recognition.
CSPG4	Melanoma, TNBC and other CSPG4-positive tumors; predominantly advanced/metastatic experimental settings	Cell-surface overexpression; promotes integrin-, Src- and FAK-associated survival, migration and invasion [77,130,131,132,133]	Monoclonal antibodies, ADC/nanoparticle conjugates, immunotoxins and TRAIL fusion proteins [134,135,136,137,138]	Evidence for the approaches discussed here is predominantly preclinical. Target internalization is particularly relevant for immunotoxins and intracellular payload-delivery strategies [137].
Heparanase	Multiple myeloma and solid tumors characterized by increased heparanase activity, invasion and ECM remodeling	HS cleavage releases matrix-bound growth factors and promotes angiogenesis, invasion, SDC-1 shedding and tumor–stroma signaling [74,105,139,140,141,142]	Heparin-related compounds, polysulfated saccharides/HS mimetics, nucleic acid-based inhibitors and neutralizing antibodies [140,143,144,145,146,147]	Predominantly experimental/preclinical approaches. Therapeutic activity is based mainly on inhibition of HS cleavage and signaling-niche remodeling rather than receptor internalization. LMWH effects should not be interpreted as exclusively heparanase-mediated [143,144].

## Data Availability

No new data were created or analyzed in this study. Data sharing is not applicable.
